# AFM negatively regulates the infiltration of monocytes to mediate sepsis-associated acute kidney injury

**DOI:** 10.3389/fimmu.2023.1049536

**Published:** 2023-01-30

**Authors:** Caiyun Guo, Youling Fan, Jiurong Cheng, Yingdong Deng, Xiangsheng Zhang, Yanna Chen, Huan Jing, Wenjun Li, Pei Liu, Jiaqi Xie, Wenjun Ning, Hongtao Chen, Jun Zhou

**Affiliations:** ^1^ Department of Anesthesiology, The Third Affiliated Hospital, Southern Medical University, Guangzhou, China; ^2^ Department of Anesthesiology, The First People's Hospital of Kashgar, Xinjiang, China; ^3^ Department of Anesthesiology, The Second People’s Hospital of Panyu, Guangzhou, China; ^4^ Department of Anesthesiology, Guangzhou Eighth People’s Hospital, Guangzhou Medical University, Guangzhou, Guangdong, China

**Keywords:** SA-AKI, AFM, monocyte, WGCNA, immunity

## Abstract

**Background:**

Sepsis is organ dysfunction due to the host’s deleterious response to infection, and the kidneys are one of the organs damaged in common sepsis. Sepsis-associated acute kidney injury (SA-AKI) increases the mortality in patients with sepsis. Although a substantial volume of research has improved the prevention and treatment of the disease, SA-SKI is still a significant clinical concern.

**Purpose:**

Aimed to use weighted gene co-expression network analysis (WGCNA) and immunoinfiltration analysis to study SA-AKI-related diagnostic markers and potential therapeutic targets.

**Methods:**

Immunoinfiltration analysis was performed on SA-AKI expression datasets from the Gene Expression Synthesis (GEO) database. A weighted gene co-expression network analysis (WGCNA) analysis was performed on immune invasion scores as trait data, and modules associated with immune cells of interest were identified as hub modules. Screening hub geneset in the hub module using protein-protein interaction (PPI) network analysis. The hub gene was identified as a target by intersecting with significantly different genes screened by differential expression analysis and validated using two external datasets. Finally, the correlation between the target gene, SA-AKI, and immune cells was verified experimentally.

**Results:**

Green modules associated with monocytes were identified using WGCNA and immune infiltration analysis. Differential expression analysis and PPI network analysis identified two hub genes (*AFM* and *GSTA1*). Further validation using additional AKI datasets GSE30718 and GSE44925 showed that *AFM* was significantly downregulated in AKI samples and correlated with the development of AKI. The correlation analysis of hub genes and immune cells showed that *AFM* was significantly associated with monocyte infiltration and hence, selected as a critical gene. In addition, Gene single-enrichment analysis (GSEA) and PPI analyses results showed that *AFM* was significantly related to the occurrence and development of SA-AKI.

**Conclusions:**

*AFM* is inversely correlated with the recruitment of monocytes and the release of various inflammatory factors in the kidneys of AKI. *AFM* can be a potential biomarker and therapeutic target for monocyte infiltration in sepsis-related AKI.

## Introduction

Sepsis is an organ dysfunction caused by a dysregulated infection response in a patient (1). The kidney is one of the organs most commonly affected by sepsis, and kidney damage can lead to multiple organ dysfunction through long-term effects (2). In intensive care patients, SA-AKI is a common complication that increases the risk of chronic kidney disease and mortality is extremely high (3). Therefore, understanding the occurrence and development mechanism of SA-AKI is significant for the treatment of sepsis patients and for preventing long-term complications.

It has been suggested that SA-AKI develops and occurs because of a variety of complex mechanisms, inflammatory immune dysregulation plays a crucial role in the occurrence and development of SA-AKI, among other factors (4). AKI often presents a hyperinflammatory state accompanied by elevated systemic cytokine levels, such as IL-6 and TNF-α (5). The damaged kidney in SA-AKI, is a major source of inflammatory chemokines, cytokines, and reactive oxygen species, closely related to the damage of various organs in the system during the progression of sepsis (6). In the early stages of AKI, high levels of these inflammatory factors lead to pro-inflammatory, neutrophil activation, and endothelial dysfunction (7). Previous studies have shown that among immune cells involved in kidney inflammation, neutrophils (recruited during the acute phase), Ly-6C+ (inflammation) monocytes, and resident macrophage populations initiate inflammatory processes that affect acute inflammation or profibrotic changes (8). However, a persistently high inflammatory state inhibits immune system function and clearance of infection, and studies suggest that AKI may attenuate the pro-inflammatory effects of neutrophils and lead to impaired monocyte function (9). In contrast, neutrophil function suppression is more intense in patients with SA-AKI. Therefore, the inflammatory immune response is a crucial entry point for treating SA-AKI. A recent study using single-cell sequencing technology identified inflammatory macrophage subsets as therapeutic targets for alleviating AKI (10). The relationship between SA-AKI and the inflammatory immune response is complex, so we aimed to screen for therapeutic targets by studying the infiltration of immune cells in the SA-AKI-induced kidney damage and provide novel ideas for diagnosing and treating the disease.

## Materials and methods

### RNA expression data

**Figure 1:**
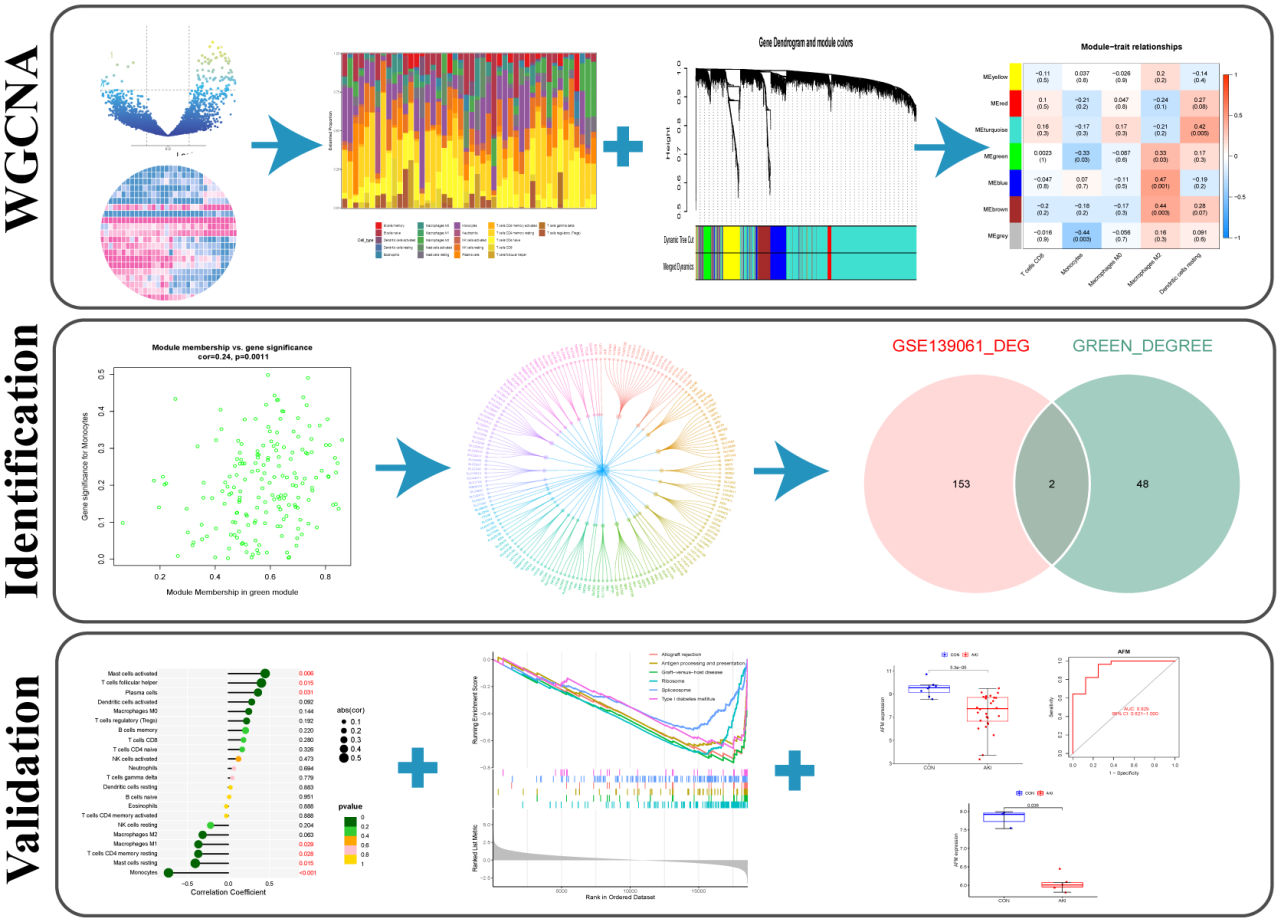
The workflow of the study.

The research strategy of the study is illustrated in **Figure 1**.

Three gene expression datasets (GSE139061, GSE30718, and GSE44925) were downloaded from Gene Expression Omnibus (GEO; https://www.ncbi.nlm.nih.gov/geo/).

The GSE139061 dataset includes 39 AKI kidney biopsy samples and 9 nephrectomy samples. The AKI kidney biopsy samples were obtained from patients with confirmed sepsis and pathological diagnosis consistent with AKI. Samples from nephrectomy surgeries were obtained from the University of Michigan’s Renal Precision Medicine Program, and Illumina HiSeq 4000 was used for sequencing (11). GSE308718 dataset includes 28 renal biopsy samples from 26 post-transplant AKI patients with an average estimated glomerular filtration rate (eGFR) of 26 mL/min at biopsy and 11 original protocol biopsy samples from stable transplants without histological abnormalities (12). The mean estimated glomerular filtration rate (eGFR) at biopsy was 51.2 mL/min. This dataset was generated using the GPL570 [HG-U133_Plus_2] Affymetrix Human Genome U133 Plus 2.0 Array (Affymetrix, Santa Clara, CA, USA). The GSE44925 dataset was derived from Affymetrix’s Mouse Gene 1.0 ST Array [transcript (gene) version] platform based on GPL6246. Finally, five AKI samples (GSM1093979, GSM1093980, GSM1093981, GSM1093982, and GSM1093983) and three normal samples (GSM1093973, GSM1093974, and GSM1093975) were selected from GSE44925 (13).

### Gene co-expression network analysis of AKI

The R package of weighted gene co-expression network analysis (WGCNA) was used to construct the weighted co-expression network (14). As a result of these calculations, we were able to cluster the samples and eliminate the outliers based on the average linkage and Pearson’s correlation coefficients. Next, an adjacency matrix was built, and a soft threshold β was selected to construct a scale-free network. In addition, a topological overlap matrix (TOM) was derived from the adjacency matrix. Finally, a hierarchical clustering tree was constructed using the dynamic clipping tree algorithm and the network modules were identified.

### Evaluation of immune cell infiltration

CIBERSORT is an analytical algorithm that uses gene expression data to estimate the abundance of each cell type in mixed cell populations (15). The infiltration fraction of the leukocyte signature matrix (LM22) was calculated using the R package “CIBERSORT” in this study, and the result was shown as a heatmap. In addition, the results between groups (CON-AKI) were compared, immune cell types with significant differences between groups were screened (P<0.05), and the fraction of immune cells in each sample was used as the trait data for WGCNA.

### Identification of hub modules and genes

Among the six modules obtained by WGCNA, those with a high correlation with the immune cells of interest were screened as hub modules by correlation coefficient and significance P value. Then, the protein-protein interactions (PPIs) of hub module genes were analyzed using STRING database (STRING; https://string-db.org/) (16). The PPI network determined the number of protein nodes applied to the central node. Subsequently, these results were combined using Cytoscape (Cytoscape_v3.9.0; https://cytoscape.org/) (17). Differential expression analysis was performed on dataset GSE139061, screened for Adjusted P value (P.adj) <0.05 and |log(Fold Change)| (|logFC|)>1. The 30 genes with the most significant differential multiples in the up-regulated and down-regulated genes among the differential genes were selected to display on the heat map. The differentially expressed genes were intersected with the central node genes to obtain the hub genes.

### Validation of hub genes

Datasets GSE30718 and GSE44925 were downloaded from the GEO database to determine the differential expression of hub genes in AKI kidney and healthy kidney tissue from human and mouse species. Reliability of receiver operating characteristic (ROC) curve test for diagnosing AKI central genes using the R package “pROC” (18).

### Identification of immune characterization

Using the R package “ggstatsplot” (19), the correlation of hub genes with relevant immune cells was analyzed by scatter plots of hub gene expression and immune cell infiltration levels. P<0.05 indicated statistical significance.

### Gene single-enrichment analysis of target genes

GSEA is a computational method to determine whether a fundamentally defined set of genes is statistically significantly different between two biological states (20). According to the median of the target gene expression, the samples were divided into two groups, and the results of GSEA were statistically significant when P.adj<0.05 and q<0.05. Finally, the enrichment pathways were visualized using the R packages “ggplot2” (21) and “clusterProfiler” (22).

### Cell culture

Human monocytic leukemia cell line (THP-1) was purchased from the Procell Life Science&Technology Co.,Ltd (Wuhan, China) and cultured in THP-1 special medium [CM-0233, Procell Life Science&Technology Co.,Ltd (Wuhan, China)] at 37°C in a 5% CO_2_ incubator. In all experiments, the cells were cultured in 6-well plates, treated with 1 μg/mL LPS [lipopolysaccharide, Sigma-Aldrich (Shanghai) Trading Co.,Ltd. (Shanghai, China)] for 24 h, and harvested; then, total RNA was extracted.

### Animals and treatments

Male wild-type C57BL/6J mice (8–10-weeks-old) were purchased from SiPeiFu Biotechnology Co., Ltd (Beijing, China), housed under standard conditions (12 h light/dark cycle) at constant temperature (22 ± 2°C) and humidity (60%), with given free access to food and water.

After 2 weeks, 12 mice were divided into two groups randomly (n=6): control and LPS. Mice in the LPS group were injected LPS (10 mg/kg) intraperitoneally, while in the control group, mice received an equal volume of saline. The blood and kidney tissues of mice were collected 12 h after LPS treatment.

In the unilateral ischemia-reperfusion (UIRI) model, after a midline abdominal incision, the left renal pedicle was dissected and clamped using microvascular clamps for 45 min at 37 °C. After ischemia, the clamps were released for reperfusion. On day 2 post-UIRI, mice were killed, and the blood and kidneys were collected. The untreated right kidney served as a control group. In addition, the blood of healthy wild-type mice was taken as a control for detecting serum creatinine urea nitrogen in UIRI-induced AKI mice.

The animal experiments were approved by the Animal Protection Committee of the Third Affiliated Hospital of Southern Medical University and conformed to the ethical standards of the Animal Ethics Committee of the Third Affiliated Hospital of Southern Medical University (Guangdong, China).

### Assessment of renal function

Mouse whole blood was coagulated at room temperature for 2 h, and serum was collected from the supernatant obtained by centrifugation at 2000 g for 20 min. Blood urea nitrogen (BUN) and serum creatinine (SCr) were determined using a urea detection kit and a Cr detection kit (C013-2-1 and C011-2-1, Nanjing Jiancheng Bioengineering Institute, Nanjing, China).

### Kidney injury assessment

Kidney coronal sections were subjected to imaging analysis. The specimens were fixed in 4% paraformaldehyde for at least 24 h before paraffin embedding. Then, hematoxylin-eosin (HE) staining was then performed on the paraffin block in 4-μm sections (23). There were five signs of tubular injury: dilated tubules, atrophy of tubules, formation of tubular casts, shedding of tubular epithelial cells, disappearance of brush borders, and the thickening of the tubular basement membrane. The tubular injury score was calculated semiquantitatively as follows: score 0: no tubular injury; score 1: <10% tubular injury; score 2: 10–24% tubular injury; score 3: 25–49% tubular injury; score 4: 50–74% tubular damage; score 5: damaged tubules ≥75%. Each specimen was randomly examined under 200× magnification in ten fields (24).

### RNA extraction and quantitative real time polymerase chain reaction

Total RNA was extracted from whole kidney tissue and monocytes using TRIzol (R0016; Beyotime, Shanghai, China). On a NanoVue (GE Healthcare, USA), absorbance at 260 nm (A260) and 280 nm (A280) were calculated to determine concentration and quality of the extracted RNA. Using a HiScript II Q RT SuperMix for qPCR kit (R222-01, Vazyme Biotech Co., Ltd. China), RNAs whose A260/A280 ratios are 1.8 to 2.0 are suitable for subsequent complementary DNA (cDNA) synthesis. The PCR amplification was performed using the ChamQ Universal SYBR qPCR Master Mix kit (Q711-02, Vazyme Biotech). Each gene was amplified for 40 cycles. We tested all samples three times, and analyzed their melting curves. The target gene mRNA expressions were normalized to that of *GAPDH* using the 2-^ΔΔCt^ method. The PCR primers were synthesized by Tsingke Biotechnology Co., Ltd, and the sequences are listed in **Table 1**.

**Table 1:** Primers for real time-quantitative PCR.

Species	Genes	Primer sequences	
**Mouse**	AFM	Forward	CCGGACAAGTTCTTTGCTGA
		Reverse	AGAGCTGCCACCATTTCCTT
	CCL2	Forward	TAAAAACCTGGATCGGAACCAAA
		Reverse	GCATTAGCTTCAGATTTACGGGT
	GAPDH	Forward	GGCCTCCAAGGAGTAAGAAA
		Reverse	GCCCCTCCTGTTATTATGG
	NF-κb	Forward	GAGTCACGAAATCCAACGCAG
		Reverse	CCAGCAACATCTTCACATCCC
	IL-18	Forward	GCCATGTCAGAAGACTCTTGCGTC
		Reverse	GTACAGTGAAGTCGGCCAAAGTTGTC
	IL-6	Forward	TAGTCCTTCCTACCCCAATTTCC
		Reverse	TTGGTCCTTAGCCACTCCTTC
	TNF-α	Forward	AGGTTCTGTCCCTTTCACTCACTGG
		Reverse	AGAGAACCTGGGAGTAGACAAGGTA
**Human**	AFM	Forward	CAGACACCTTCTTTGCGAAGT
		Reverse	GCGTAACGGTAACAACCTGG
	CCL2	Forward	AGCAGCAAGTGTCCCAAAGA
		Reverse	TTGGGTTTGCTTGTCCAGGT
	GAPDH	Forward	GAGAAGGCTGGGGCTCATTT
		Reverse	AGTGATGGCATGGACTGTGG
	NF-κb	Forward	TGCAGCAGACCAAGGAGATG
		Reverse	CCAGTCACACATCCAGCTGTC
	IL-18	Forward	TGCATCAACTTTGTGGCAAT
		Reverse	CAGCTCTGGCTTGTTCCTCA
	IL-6	Forward	CCAGTACCCCCAGGAGAAGA
		Reverse	CAGCTCTGGCTTGTTCCTCA
	TNF-α	Forward	CTGGAAAGGACACCATGAGCA
		Reverse	TCTCTCAGCTCCACGCCATT

### Western blot analysis

Mouse kidney tissues and monocyte were dissected and homogenized in radio immune precipitation (RIPA Lysis buffer) lysis buffer (P0013B, Beyotime Biotechnology, China) containing protease inhibitors (FD1001, FUDE Biological Co., Ltd., Hangzhou, China). After centrifugation at 4°C at 12000 rpm for 15 min, the supernatant was collected, and the protein concentration was determined using the BCA Protein Detection Kit (23225, Thermo-Scientific). Bovine serum albumin is the standard. An equal amount of protein lysate is loaded directly on 10 SDS-PAGE and transferred to a polyvinylidene fluoride (PVDF) membrane for western blotting (0.2 μm) (ISEQ00010, MilliporeSigma). Plug the membrane with 5% skim dry milk in Tris-buffered saline and 0.1% Tween-20 (TBS-T) for 1 h at room temperature, followed by overnight incubation with the designated primary antibody at 4°C. After rinsing three times every 5 min with TBS-T, dilute with goat anti-rabbit IgG (1:5000; Signalway Antibody LLC) incubate for 1 h.

Western blotting was visualized using FDbio-Dura Ecl luminescent solution (FD8020, FUDE Biological Co., Ltd., Hangzhou, China) and was visualized under the Tanon-5200 Chemiluminescent Imaging System (Tanon Science and Technology, Beijing, China). Density analysis using ImageJ6.0 software (National Institutes of Health, Bethesda, MD, USA).

### Enzyme-linked immunosorbent assay

IL-6, IL-18, and TNF-α levels in kidney tissues were measured using the IL-6 mouse ELISA kit (MM-1011M2, Jiangsu, China), IL-18 mouse ELISA kit (MM-0906M2, Jiangsu, China), and TNF-α mouse ELISA kit (MM-0132M2, Jiangsu, China), respectively.

### Statistical analysis

Statistical analysis was performed using GraphPad Prism (GraphPad Prism 9; GraphPad Software, Inc.). The data conformed to a normal distribution. Unpaired Student’s t-test was used to analyze the differences between the two groups. P<0.05 indicated a statistically significant difference.

## Results

### Construction of gene co-expression network

**Figure 2:**
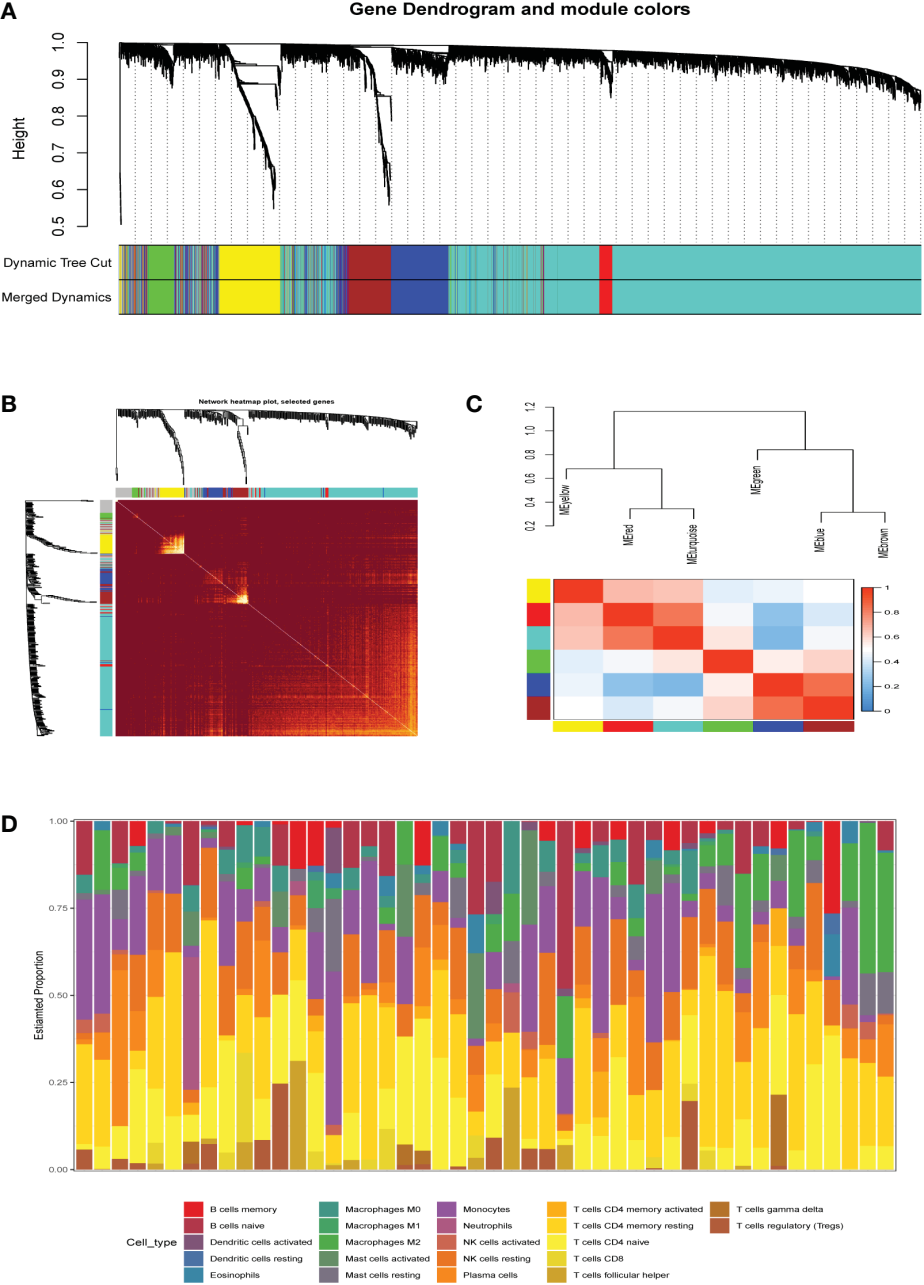
**(A)** Clustering dendrogram of genes, with dissimilarity based on topological overlap, together with assigned merged module colors and the originalmodule colors. **(B)** The heat map depicts the topological overlap matrix (TOM) of all genes in the analysis. Gene dendrograms and module assignmentsare also shown on the left and top. **(C)** Genes are classified into different modules by hierarchical clustering, different colors below the hierarchicalclustering tree represent different modules. **(D)** Correlation heatmap of infiltration of immune cells in normal and AKI renal tissues.

A co-expression network of 5000 gene expression values for 46 samples was constructed using the R software package “WGCNA” (14). Consequent to clustering, two outlier samples were removed (**Supplementary Figure 1**). In this study, we selected the soft threshold β=6 (scale-free R2 = 0.85) to construct a scale-free network and used the dynamic clipping tree algorithm for clustering to construct a hierarchical clustering tree (**Figures 2A, B**). Each leaf of the tree represents a gene. Then, the genes of the expression data were combined to form the branches of the tree, representing a gene module; a total of six modules were identified for subsequent analysis (**Figure 2C**).

### Identification of hub modules and genes

**Figure 3:**
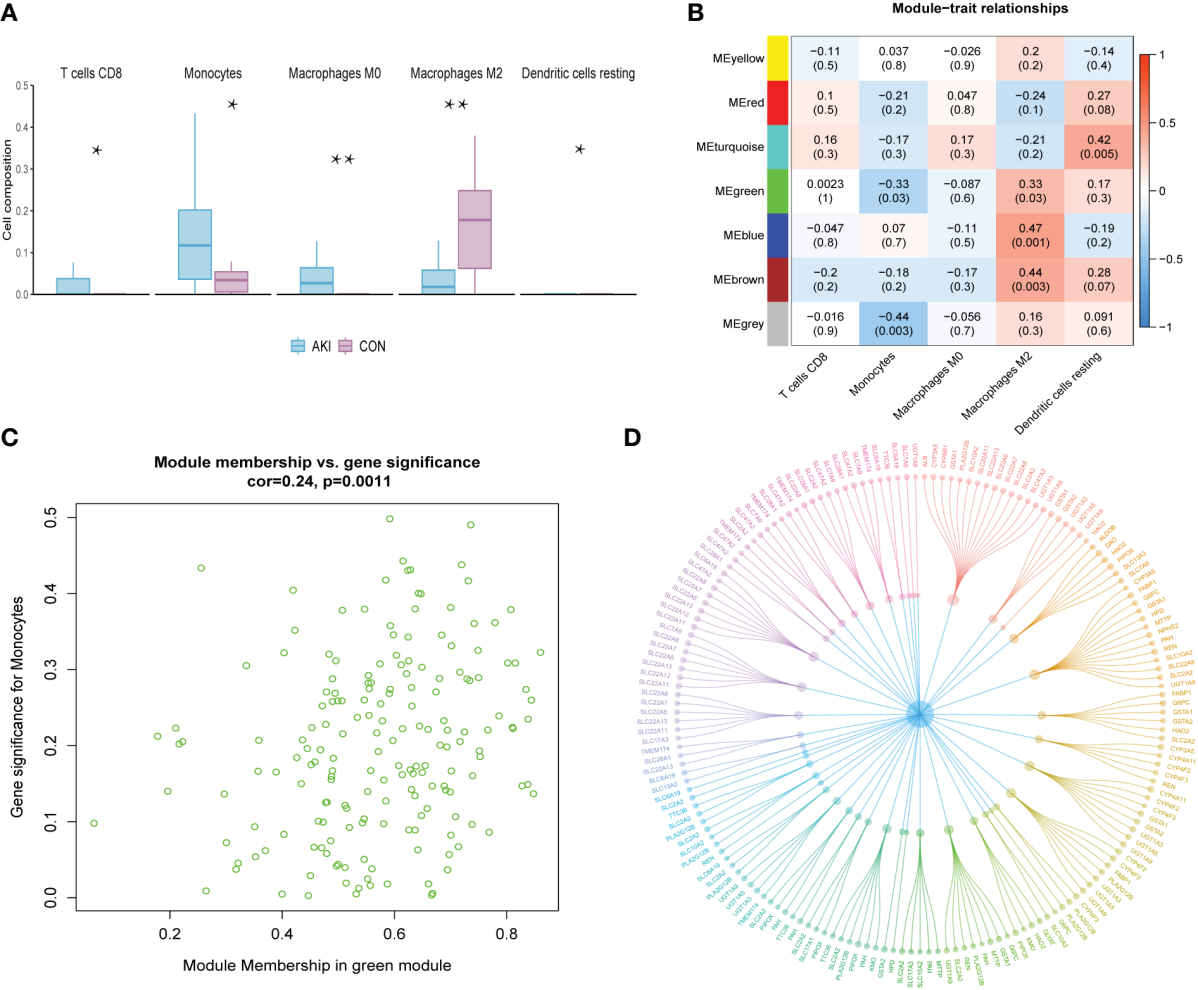
**(A)** The grouped box plot shows immune cells with significantly different immune infiltration scores between the control and experimental groups.**(B)** Heatmap shows the correlation between module eigengenes and immune cells. **(C)** Scatter plot of green module eigengenes. **(D)** PPI network ofgreen module genes. *p < 0.05, **p < 0.01.

The R package “CIBERSORT” was used to calculate the infiltration scores of various immune cells in the gene expression matrix (25). The immune cell infiltration was visualized with a heatmap (**Figure 2D**). The differences in immune cell infiltration scores between the AKI and normal kidney groups were compared. T cells CD8, monocytes, macrophages M0 and M2, and dendritic cells resting were selected according to P<0.05 (**Figure 3A**). The five types kinds of immune cells and their infiltration scores in each sample were used as trait data for weighted correlation network analysis. The correlation between the module signature genes and the infiltration of five types of immune cells was displayed on a heat map (**Figure 3B**). The green modules correlated with both monocytes (R2=-0.33, P=0.03) and M2 macrophages (R2 = 0.33, P=0.03), while the blue (R2 = 0.47, P=0.001) and brown modules (R2 = 0.41, P=0.003) were associated with M2 macrophages. The turquoise-colored module was associated with dendritic cells resting (R2 = 0.42, P=0.005). Herein, we selected the green module as the hub. A scatter plot of the distribution of genes in the hub module is shown in **Figure 3C**. The hub module genes were analyzed using the STRING database for PPI analysis and were sorted according to the number of nodes. The top 50 genes with the number of nodes were screened as the central node (**Figure 3D**). These 50 genes intersected with the differential genes of the dataset, and two hub genes, *AFM* and *GSTA1* were obtained.

### Screening of differentially expressed genes

**Figure 4:**
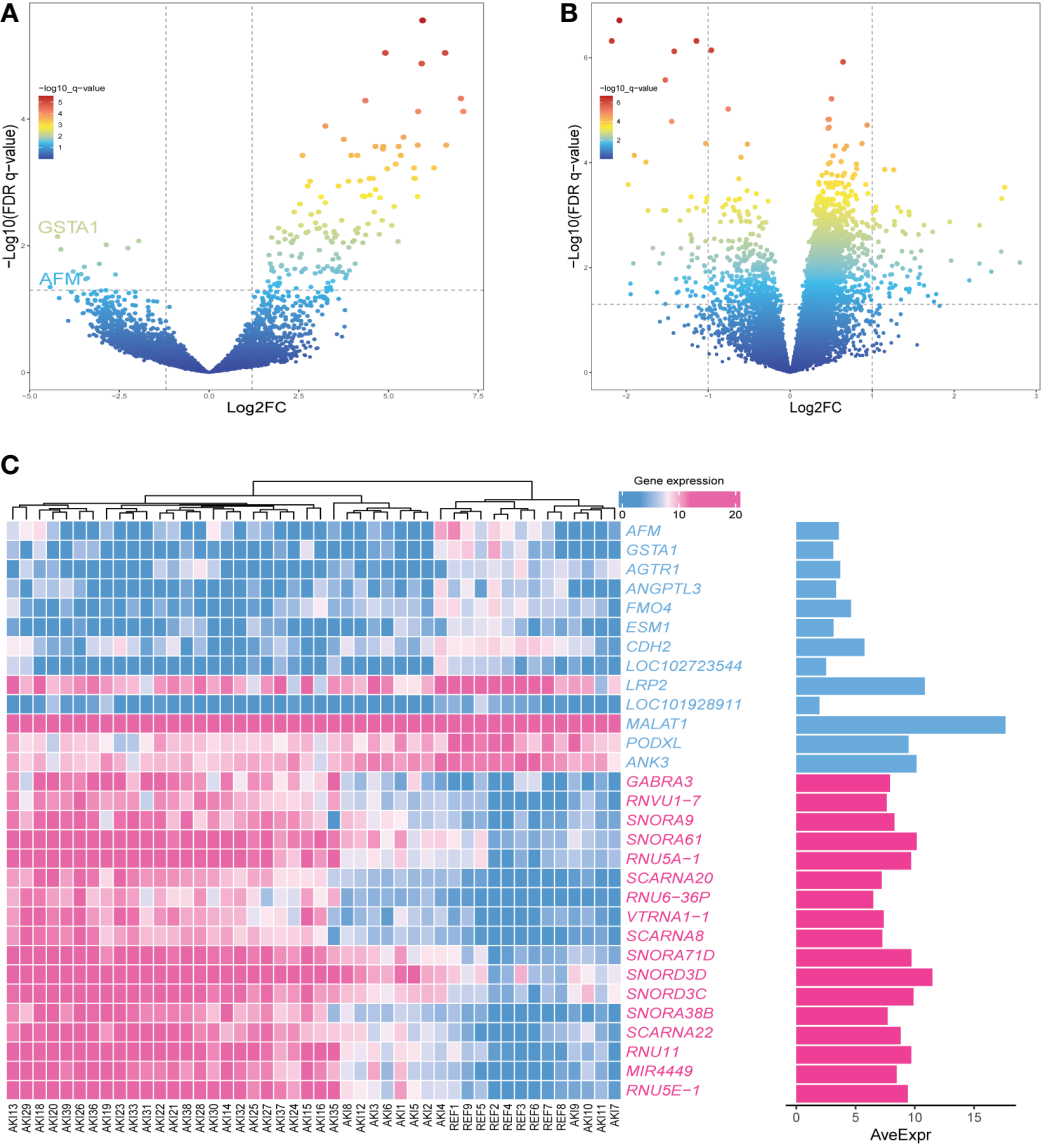
**(A)** The volcano plot showing the differential gene expression (fold change ≥ 1; FDR < 0.05) between control and AKI group in GSEGSE139061 dataset.**(B)** The volcano plot showing the differential gene expression (fold change ≥ 1; FDR < 0.05) between control and AKI group in GSE30718 dataset.**(C)** Heatmap shows the most significant 30 genes in up- and down-regulation.

The R package “limma” was used to screen the DEGs of AKI and normal kidney samples in GSE139061 (26). According to P.adj<0.05, |logFC|>1, a total of 155 DEGs were screened, of which 142 were upregulated and 13 were downregulated. The volcano plots were visualized in **Figure 4A**. The 50 most significant DEGs among the upregulated and downregulated genes were displayed on a heat map (**Figure 4C**).

### Validation of hub genes

**Figure 5:**
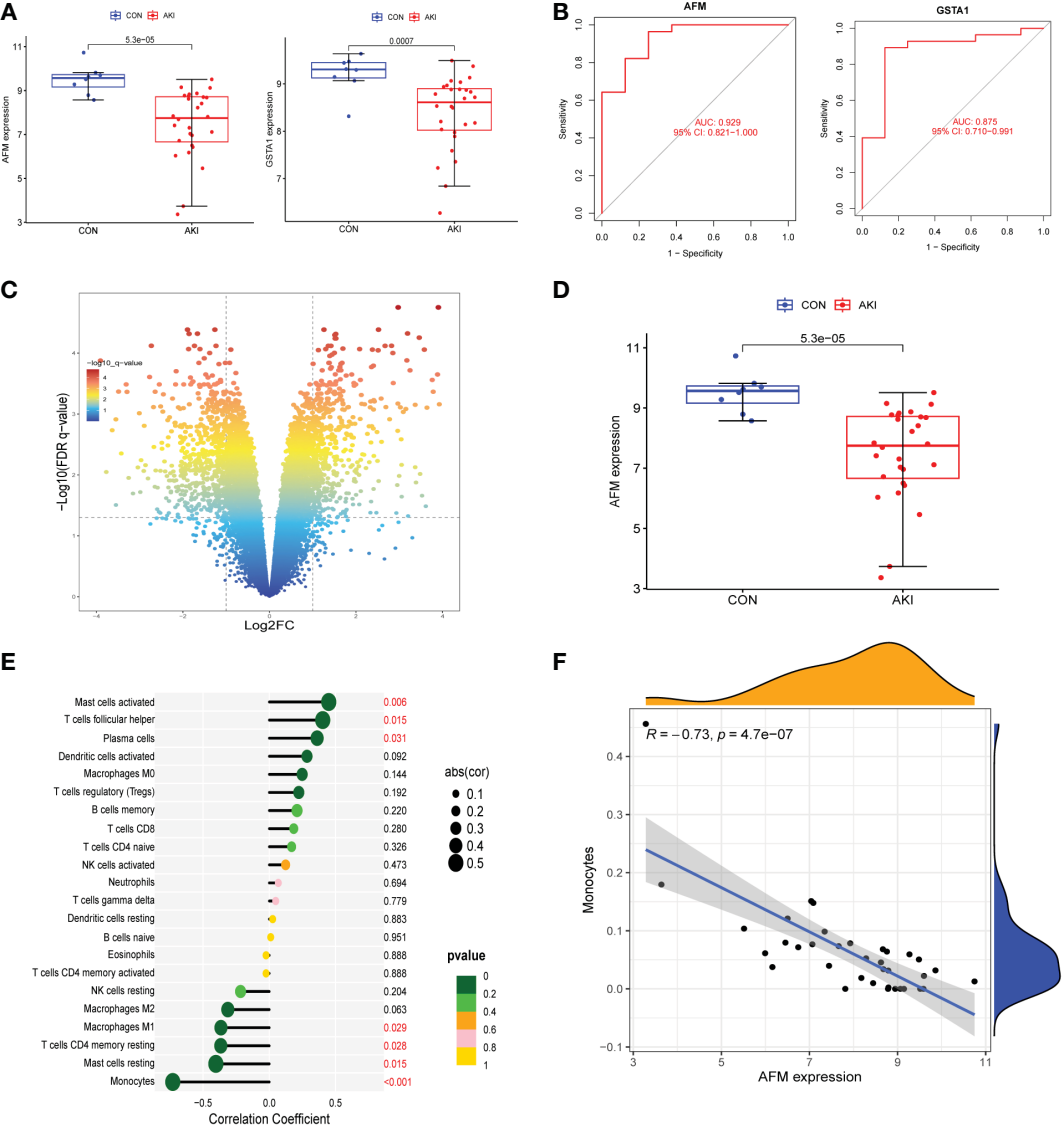
**(A)** The expression of AFM and GSTA1 were high in the AKI group in the GSE30718 dataset. **(B)** ROC curves for AFM, GSTA1. (95% confidence interval (CI), AFM: 0.817-1.000; GSTA1: 0.714-0.996.) **(C)** The volcano plot showing the differential gene expression (fold change ≥ 1; FDR < 0.05) between control and AKI group in GSE44925 dataset. **(D)** The expression of AFM and GSTA1 were high in the AKI group in the GSE44925 dataset. **(E)** The relationship between AFM expression and immune cell infiltration level; P < 0.05 was considered statistically significant. **(F)** Scatter plot of AFM expression versus level of monocyte infiltration.

The datasets GSE30718 and GSE44925 were downloaded from the GEO database. The volcano plot shows the differential expression of datasets GSE30718 and GSE44925 (**Figures 4B**, **5**C). The expression of two hub genes was downregulated in the AKI group of the dataset GSE30718 (*AFM*: P=5.3e-0.5, *GSTA1*: P=0.0007) (**Figure 5A**). The ROC curve analysis of the diagnostic value of the two genes for AKI found that the area under the curve (AUC) of the two genes was as follows: *AFM*: 0.929; *GSTA1*: 0.875. The AUC of *AFM* was larger than that of *GSTA1*, suggesting that it had a better diagnostic value (**Figure 5B**). Moreover, *AFM* was significantly underexpressed in the AKI group in dataset GSE44925 (**Figure 5D**). Next, we searched the diseases associated with AFM and GSTA1 in The Comparative Toxicogenomics Database (CTD) and found that both genes were associated with AKI (AFM: 67.18; GSTA1: 264.77). We present this result in **Table 2**. In conclusion, we selected *AFM* as the target gene for our study.

**Table 2:** Relationship to inflammation and kidney disease to key genes based on the CTD database.

Genes	Disease name	Direct Evidence	Inference score	References
AFM	Inflammation	–	115.82	103
	Fibrosis	–	79.67	62
	Kidney Diseases	–	74.39	387
	Acute Kidney Injury	*	67.18	208
GSTA1	Inflammation	–	291.13	467
	Kidney Diseases	–	264.77	671
	Acute Kidney Injury	*	167.66	627

* A gene that may be a biomarker of a disease.

### GSEA of the target gene

**Figure 6:**
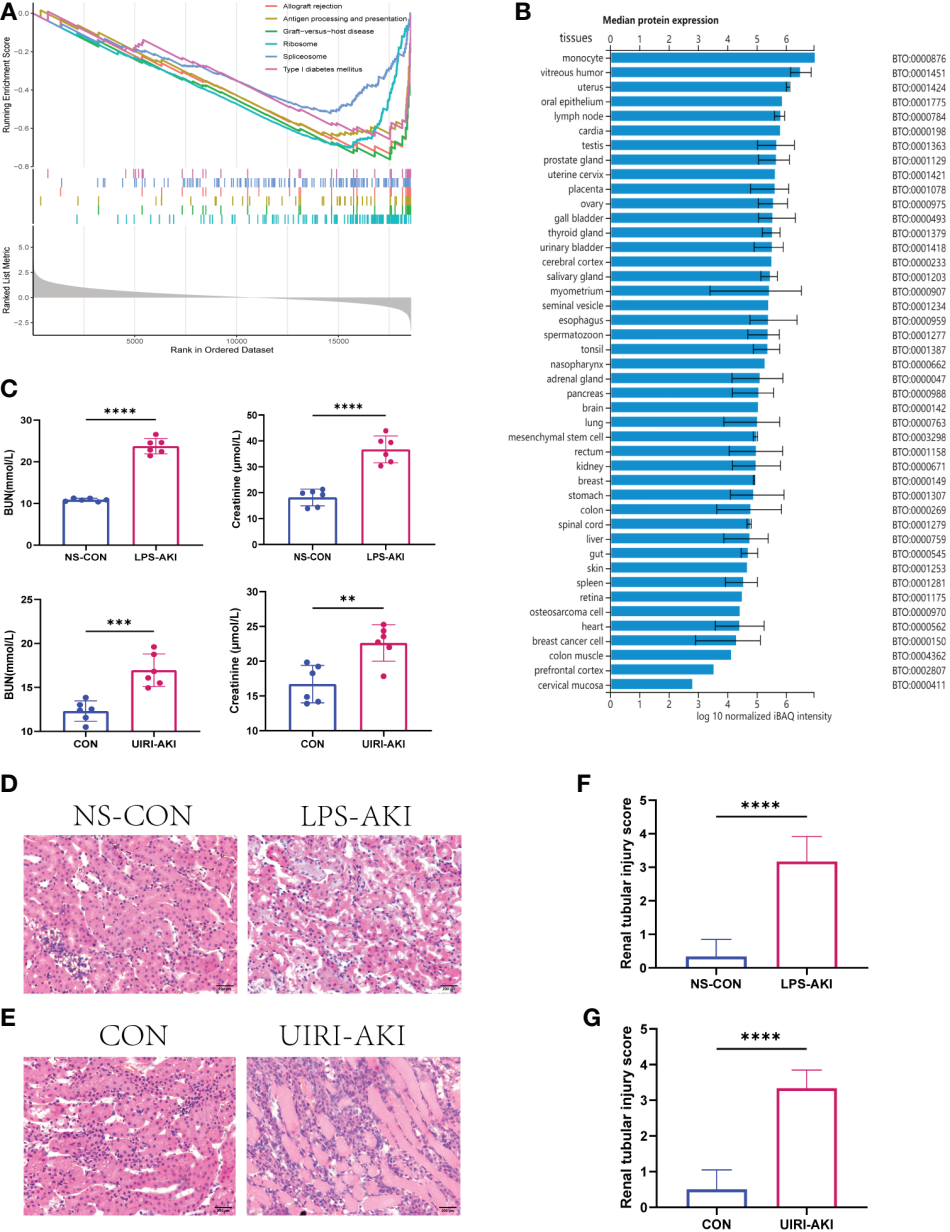
**(A)** GSEA of AFM. The first part shows the enrichment fraction broken line of six pathways, the line in the middle part corresponds to the genes of each diagram, and the third part shows the rank value distribution map of all genes. **(B)** The median protein expression of tissues or cells in the ProteomicsDB. **(C)** BUN and SCr values in two AKI mouse models (LPS-AKI; UIRI-AKI). **(D, E)** HE staining showed deterioration of the histological features of the renal cortex in two AKI models (LPS-AKI; UIRI-AKI). **(F, G)** The renal tubular injury score of two AKI moudules. **p < 0.01, ***p < 0.001, ****p < 0.0001.

The samples in the dataset GSE139061 were divided into the high- group and low-expression groups according to the median expression of *AFM*. The pathway enrichment analysis identified 118 significantly enriched pathways (P.adj<0.05, q<0.05). The six enriched pathways with the highest NES in the low-expression group included the immune-related pathways (graft-versus-host disease, allograft rejection, type I diabetes, and antigen processing and presentation), while the high-expression group was not enriched for the immune-related pathways (**Figure 6A**). The specific information of the six enrichment pathways with the highest NES in the high and low expression groups is shown in **Table 3**. The enrichment pathways were visualized using the R packages “ggplot2” and “clusterProfiler.”

**Table 3:** Single-gene GSEA of AFM.

	ID	setSize	NES	p.adjust
Head	hsa00053	Ascorbate and aldarate metabolism	0.709402	0.000136
	hsa04614	Renin-angiotensin system	0.694034	0.000407
	hsa04977	Vitamin digestion and absorption	0.679851	0.000678
	hsa00982	Drug metabolism - cytochrome P450	0.655219	0.00012
	hsa00591	Linoleic acid metabolism	0.654396	0.000807
	hsa00650	Butanoate metabolism	0.654048	0.000131
Tail	hsa05310	Asthma	-0.64122	0.00078
	hsa04940	Type I diabetes mellitus	-0.65781	0.000463
	hsa05320	Autoimmune thyroid disease	-0.66866	0.00044
	hsa03010	Ribosome	-0.70195	0.001427
	hsa05330	>Allograft rejection	-0.73373	0.000428
	hsa05332	Graft-versus-host disease	-0.76277	0.000428

### Significant negative correlation between AFM and monocyte infiltration

To investigate the correlation between *AFM* gene and immune cells, we analyzed the expression data of *AFM* genes in the GSE30718 dataset. The results showed that the expression value of this *AFM* was negatively correlated with the infiltration levels of monocytes, mast cells resting, T cells CD4 memory resting, and macrophages M1; the highest correlation was with monocytes (R=-0.73) (**Figure 5E**). The scatter plots of *AFM* expression and monocyte infiltration levels are shown in **Figure 5F**. Also, the expression of *AFM* in various tissues or cells was queried in the ProteomicsDB database (ProteomicsDB; https://www.proteomicsdb.org/), and it was found to be highly expressed in monocytes (**Figure 6B**).

### AFM is downregulated in mice with AKI

In this study, we constructed two AKI mice models: the LPS-induced SA-AKI and the unilateral ischemia-reperfusion (UIRI) induced AKI.

**Figure 7:**
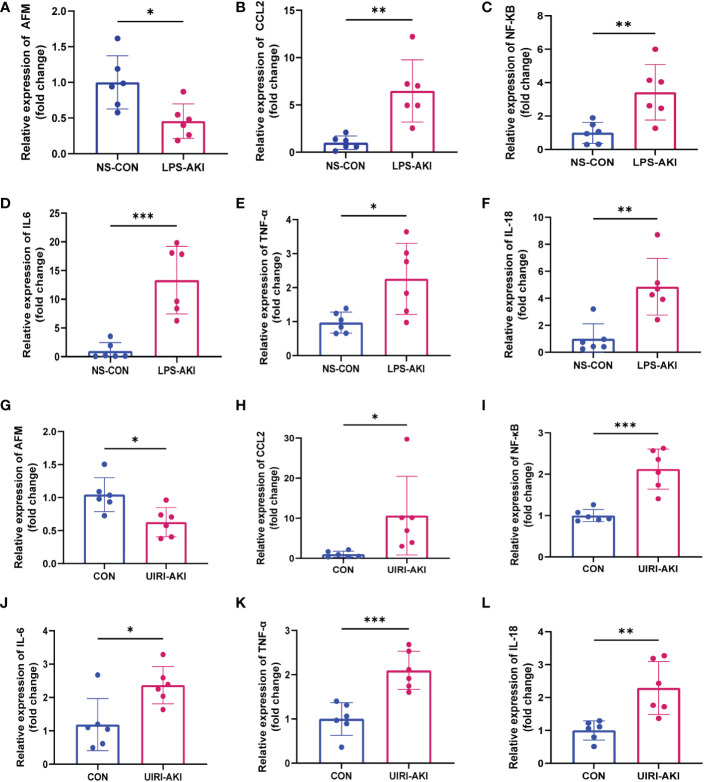
**(A–F)** Relative expression level of AFM, CCL2 and inflammatory indicators mRNA in LPS-AKI mice model. **(G–L)** Relative expression level of AFM, CCL2 and inflammatory indicators mRNA in UIRI-AKI mice model. *p < 0.05, **p < 0.01, ***p < 0.001.

**Figure 8:**
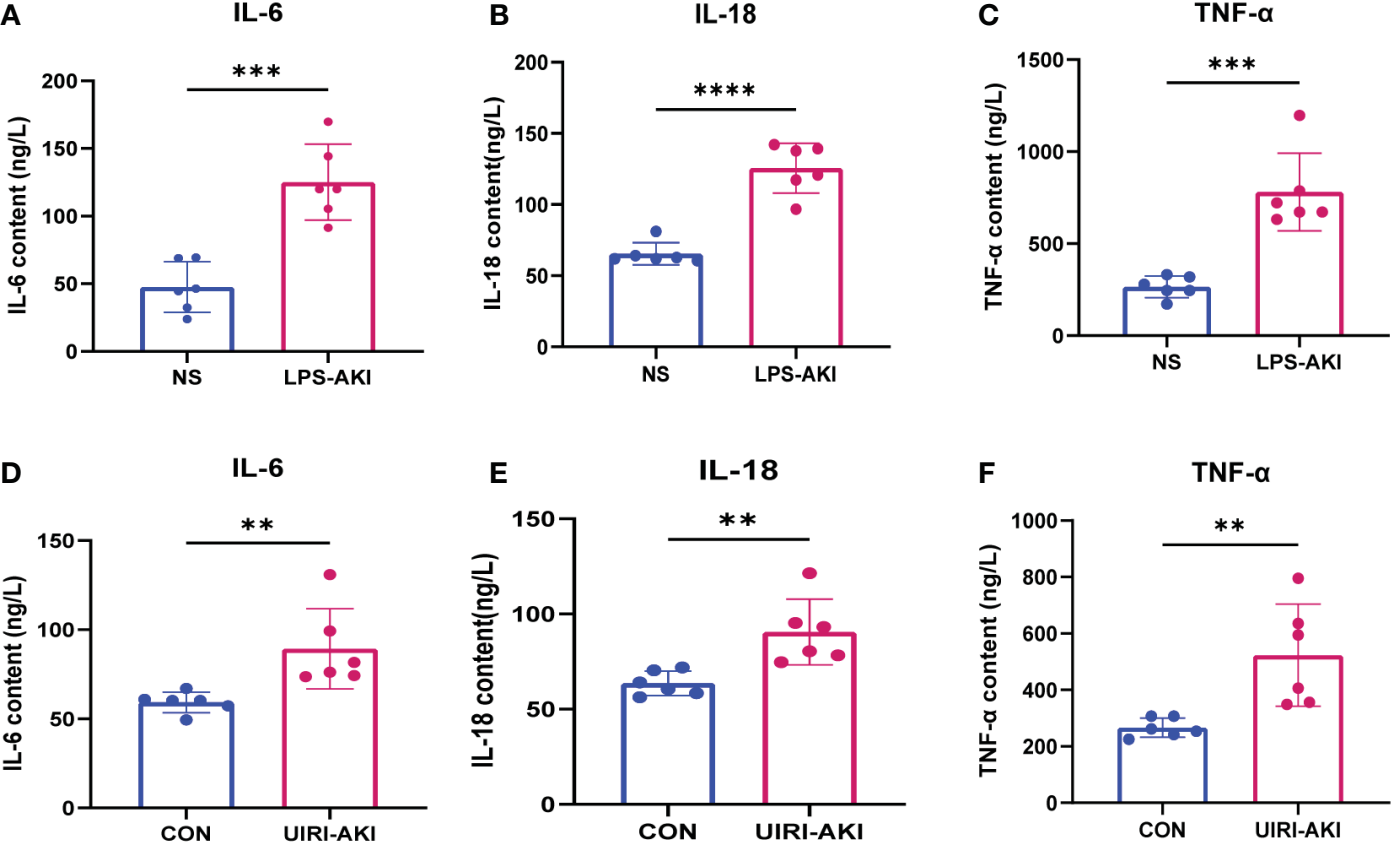
**(A–F)** ELISA analysis of IL-6, IL-18, TNF-α levels in UIRI-AKI and LPS-induced AKI models. Analyze the data using Student’s t-test. **p < 0.01, ***p < 0.001, ****p < 0.0001.

The serum BUN and creatinine levels were substantial increased in these two AKI models compared to the control group and renal function was significantly decreased (**Figure 6C**). In addition, HE staining showed deterioration of the histological features of the renal cortex in two AKI models (**Figures 6D–G**). qRT-PCR and enzyme-linked immunosorbent assay (ELISA) detected the expression of inflammatory factors (IL-6, IL-18, TNF-α) in samples (**Figures 7**, **8**). qRT-PCR detected the expression of AFM. In the AKI renal tissue, *AFM* expression was significantly downregulated, and the *CCL2* and inflammatory indicators (*NF-κB, IL-6*, *TNF-α, and IL-18*) were significantly upregulated (**Figures 7**, **8**).

### LPS inhibits the expression of *AFM* in THP-1 cells

**Figure 9:**
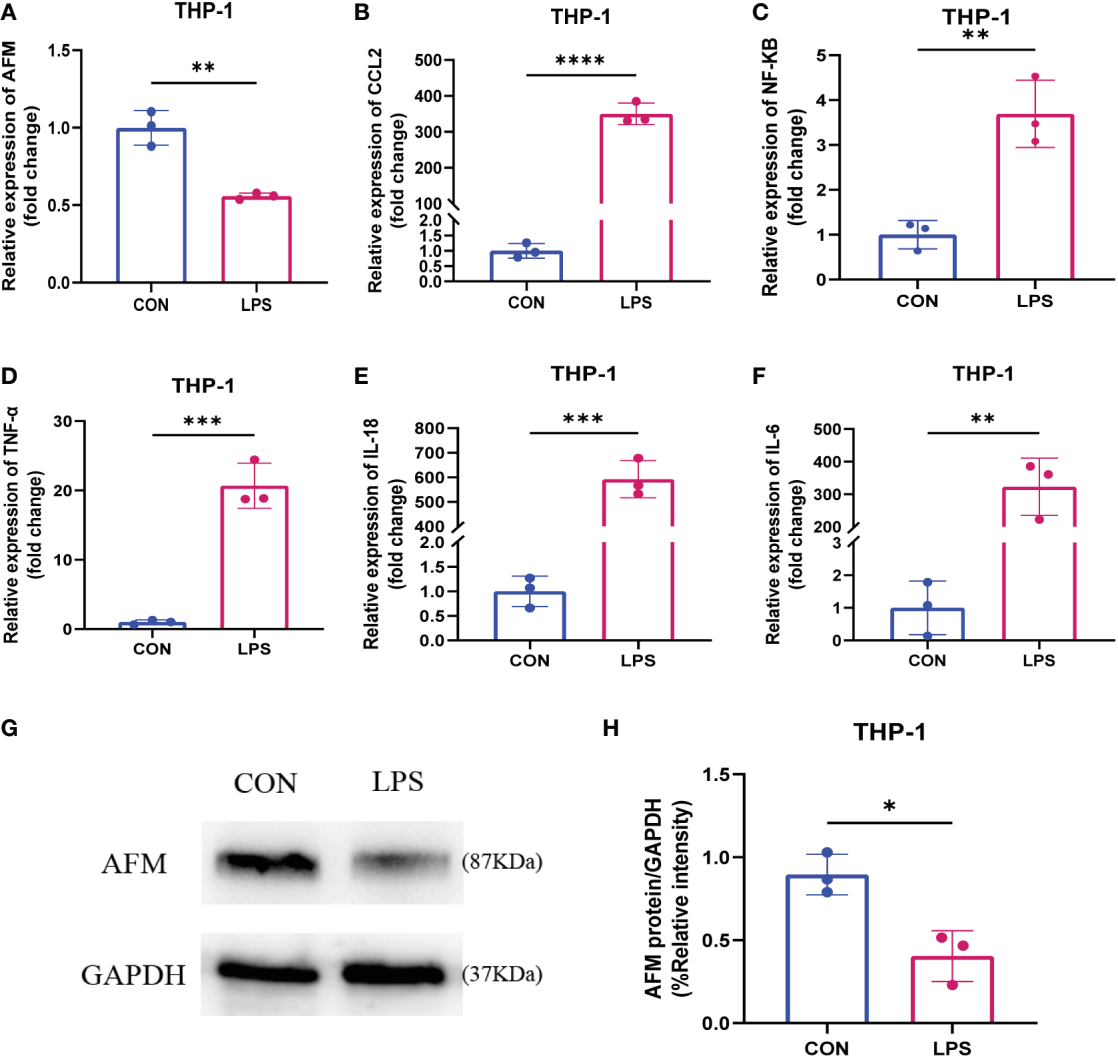
**(A–F)** Relative expression levels of AFM, CCL2 and inflammatory index mRNA in LPS-stimulated monocytes (THP-1). **(G, H)** The immunoblotting **(G)** and quantification grayscale value of AFM in THP-1 cell 24 h post-LPS stimulation **(H)**. *p < 0.05, **p < 0.01, ***p < 0.001, ****p < 0.0001.

To test whether LPS affects the expression of *AFM* in monocytes, we measured the mRNA and protein expression of *AFM* in LPS-treated THP-1 cells and found that the expression of *AFM* was significantly decreased (**Figures 9G, H**). *CCL2* is a monocyte chemokine, and the expression level of *CCL2* mRNA increased after LPS stimulation in THP-1 cells. In addition, the LPS treatment increased the mRNA levels of the inflammatory marker, *NF-κB, IL-6*, *TNF-α, and IL-18* (**Figure 9**). Based on these findings, we proposed the hypothesis that the decrease expression of *AFM* in monocytes after LPS stimulation may be associated with elevated *CCL2* expression, thereby increasing monocyte infiltration and promoting inflammation.

## Discussion

AKI is a clinical disease wherein the glomerular filtration rate decreases suddenly due to various reasons in a short time, resulting in the rapid development of water, electrolyte, acid-base balance, and systemic complications (27, 28). Clinically, the primary causes of AKI include sepsis, renal ischemia-reperfusion, and exposure to nephrotoxin, with sepsis accounting for about half of all AKI cases (29, 30). Sepsis is defined as end-organ dysfunction due to the host’s inflammatory response to infection, in which the damaged kidney, a major source of inflammatory chemokines, may have local and remote deleterious effects on the body and increase the risk of mortality in patients with sepsis (3). Several studies have shown that the occurrence and development of SA-AKI are closely related to the infiltration of various immune cells (31). AKI is characterized by inflammatory infiltration within the kidney that induces apoptosis and promotes tubular epithelial cell loss (32). In addition, recent studies have found that the circulation of inflammatory cytokines, such as IL-6 and TNF-α, is associated with an increased risk of death in patients with AKI (33). Three basic mechanisms in the development of SA-AKI include microvascular dysfunction, inflammation, and metabolic reprogramming, in which the recruitment of immune cells and the overproduction of pro-inflammatory cytokines play a critical role in developing LPS-induced SA-AKI (3, 34). Although significant efforts have been made to resolve the issues related to treatment, the early diagnosis and treatment of SA-AKI is a significant clinical problem. Therefore, the present study focused on the mechanism of immune infiltration in the development of SA-AKI and the search for new diagnostic markers and therapeutic targets through bioinformatics analysis.

In order to further explore the mechanism of immune cells in sepsis-related AKI and find novel ideas for the diagnosis and treatment, we downloaded the mouse sepsis-related AKI dataset from the GEO database and constructed a network of gene co-expression matrix and immune infiltration analysis. Five types of immune cells with significant differences in immune infiltration scores were screened between the AKI and control groups. Weighted correlation network analysis was performed using the immune infiltration score of each sample as trait data, combining the results of immune infiltration and WGCNA. Based on the analysis, we screened out *AFM* and *GSTA1* as hub genes. We downloaded the human AKI dataset and the mouse rhabdomyolysis-associated AKI dataset as external data to validate these two genes and found that *AFM* was significantly downregulated in the AKI group in both datasets. Next, the *AFM* gene was selected as a potential diagnostic and therapeutic target for SA-AKI based on the results.

*AFM*, also known as Afamin, is the fourth member of the albumin gene family and is mainly produced by the liver and expressed in the kidney (35). Some studies revealed that *AFM* has multiple binding sites for α- and γ-tocopherol and is a specific binding protein for vitamin E (36, 37). Growing evidence suggests that AFM, as a specific binding protein for vitamin E, may play a role in protecting cells from oxidative damage (38). A clinical trial assessed the strong inverse association between plasma *AFM* and inflammatory disease and biomarkers and hinted it as a potentially harmful acute-phase protein (39). On the other hand, studies on metabolic-related diseases demonstrated that plasma *AFM* significantly correlated with liver lipids, fatty liver index, and liver injury markers (40). Another study in metabolic syndrome and obesity demonstrated that *AFM* may influence the development of obesity-related oxidative stress through *via* insulin resistance (41). In addition, plasma *AFM* levels constitute an independent risk factor for gestational diabetes (42–44). The studies on kidney disease exhibited that urinary *AFM* is closely related to kidney damage and may be a potential marker of kidney damage, which is useful for the early prediction of patients with a high risk of kidney disease in patients with type 2 diabetes (45, 46). Moreover, multiple proteomic analyses have found that urinary AFM is a potential biomarker for various diseases, including membranous nephropathy, lupus nephritis, and osteoarthritis (47–49). To sum up, although several studies have revealed the molecular function of *AFM* and its correlation with various inflammatory diseases, there is still a lack of research on the role of *AFM* in the development of SA-AKI from the perspective of molecular mechanisms.

Monocyte chemokines regulate monocyte transport, namely CCL2, CCL7, CX3CL1, and various chemokine receptors. CCL2 is a well-known CC chemokine, which is not only one of the critical chemokines in regulating monocytes/macrophage migration and infiltration, but also plays a role in cancer, autoimmune diseases, bacterial and viral infections, and many kidney diseases. In some studies, CCL2 has been recognized as a novel AKI biomarker that plays a vital role in many types of AKI (50). In addition, high CCL2 levels are thought to be positively correlated with renal interstitial fibrosis and tubular atrophy (51, 52). Based on the importance of CCL2 in acute kidney injury, we selected CCL2 as the monocyte chemokine to be detected.

**Figure 10:**
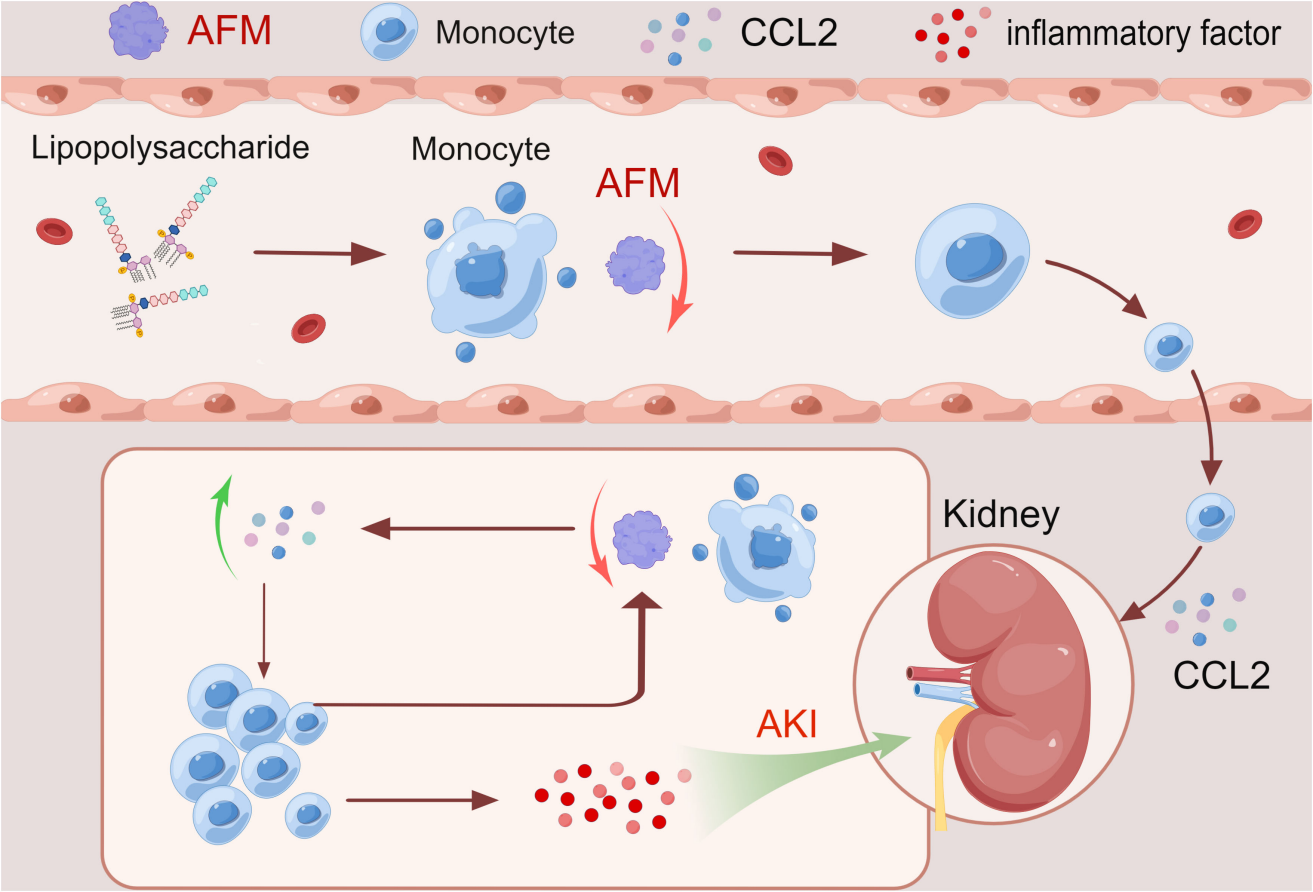
Hypothesis map was drawn by Figdraw.

In this study, we used SA-AKI and UIRI models for validation. qPCR detection showed that *AFM* in kidney tissue of SA-AKI mice induced by LPS was significantly decreased, and *CCL2* and inflammatory indexes increased considerably. Furthermore, bioinformatics analysis showed that AFM was negatively correlated with the level of monocyte infiltration. It has been previously reported that AFM can act as a chemokine for pro-osteoblasts and may stimulate osteoclastogenesis and bone resorption through Gi-coupled receptors and the Ca2+/calmodulin-dependent protein kinase (CaMK) pathway (53, 54). It suggests that AFM may be closely related to the monocyte-macrophage system’s differentiation and *in vivo* migration. Our experiments indicated that LPS-treated THP-1 decreased *AFM* expression and significantly increased *CCL2* and inflammatory markers compared with controls. Based on the above results, we propose that AFM is a potential biomarker of SA-AKI, and its downregulation may be associated with the recruitment of monocytes to the damaged kidney. Therefore, we hypothesize that AFM is downregulated in monocytes in sepsis-related acute kidney injury, which further increases monocyte recruitment and the release of various inflammatory factors in the injured kidney, thereby aggravating the inflammatory response, thereby promoting the progression of the disease. The hypothesis diagram is shown in **Figure 10**. In addition, we found that the expression of *AFM* was also significantly downregulated in UIRI-induced AKI, and the changes in *CCL2* and various inflammatory factors were similar to SA-AKI. Therefore, we propose that *AFM* negatively regulates monocyte infiltration in two models of AKI, mediating an inflammatory response that promotes disease progression. However, this study did not perform AFM gene knockout or overexpression to test this conjecture. Therefore, we will further verify this conjecture in subsequent studies. AFM may be a potential diagnostic marker for SA-AKI, and based on this, we hope that the expression of AFM in the hematuria of patients with SA-AKI can be detected in later studies as further to clarify the significance of AFM as a diagnostic marker.

In summary, AFM was significantly reduced in SA-AKI and UIRI-induced AKI and negatively correlated with monocyte infiltration. This finding suggested that AFM may affect the onset of inflammatory responses by negative regulation of monocyte infiltration, thereby regulating SA-AKI progression. These phenomena may provide novel ideas for diagnosing and treating sepsis-related AKI.

## Data availability statement

The datasets presented in this study can be found in online repositories. The names of the repository/repositories and accession number(s) can be found in the article/**Supplementary Material**.

## Ethics statement

The animal study was reviewed and approved by The Animal Protection Committee of the Third Affiliated Hospital of Southern Medical University. Written informed consent was obtained from the owners for the participation of their animals in this study.

## Author contributions

CG: Conceptualization, Methodology, Validation, Formal Analysis, Writing - Original Draft. JC: Data Curation, Writing - Original Draft. YD: Visualization. XZ: Resources. YC: Validation. HJ: Writing - Review & Editing. WL: Formal Analysis. PL: Visualization. JX: Writing - Review & Editing. WN: Validation. HC: Resources, Supervision. YF: Validation, Writing - Review & Editing, Project Administration, CG and YF contributed equally. JZ: Conceptualization, Funding Acquisition, Resources, Supervision, Writing - Review & Editing. All authors contributed to the article and approved the submitted version.
