# Supplementary material for: AFM negatively regulates the infiltration of monocytes to mediate sepsis-associated acute kidney injury
Source: Front Immunol. 2023 Jan 30;14:1049536. doi: 10.3389/fimmu.2023.1049536 (PMC9922996; doi:10.3389/fimmu.2023.1049536)
Supplement: Supplementary file 1 [file DataSheet_1.docx]

**Supplementary Table 1.** The results of PPI were obtained from the STRING database.

| **Node1** | **Node2** | **Combined_score** |
| --- | --- | --- |
| ABCC2 | CYP3A5 | 0.704 |
| ABCC2 | SLC10A2 | 0.638 |
| ABCC2 | ALB | 0.518 |
| ABCC2 | SLC22A11 | 0.584 |
| ABCC2 | SLC22A13 | 0.541 |
| ABCC2 | CYP8B1 | 0.551 |
| ABCC2 | SLC2A2 | 0.667 |
| ABCC2 | SLC47A2 | 0.558 |
| ABCC2 | GSTA1 | 0.428 |
| ABCC2 | SLC22A8 | 0.703 |
| ABCC2 | UGT1A9 | 0.725 |
| ABCC2 | PLA2G12B | 0.478 |
| ABCC2 | UGT1A3 | 0.602 |
| ABCC2 | SLC22A7 | 0.664 |
| ABCC2 | SLC22A6 | 0.703 |
| ADH6 | GSTA1 | 0.767 |
| ADH6 | UGT1A9 | 0.682 |
| ADH6 | UGT1A5 | 0.688 |
| ADH6 | UGT1A3 | 0.691 |
| ADH6 | GSTA2 | 0.773 |
| AFM | HAO2 | 0.433 |
| AGXT2 | SLC7A9 | 0.519 |
| AGXT2 | DAO | 0.873 |
| AGXT2 | ALDOB | 0.487 |
| AGXT2 | SLC13A3 | 0.662 |
| AGXT2 | HAO2 | 0.908 |
| AGXT2 | PIPOX | 0.94 |
| ALB | CYP3A5 | 0.416 |
| ALB | SLC10A2 | 0.403 |
| ALB | G6PC | 0.561 |
| ALB | REN | 0.841 |
| ALB | HPD | 0.554 |
| ALB | FABP1 | 0.828 |
| ALB | UGT1A9 | 0.412 |
| ALB | GSTA1 | 0.44 |
| ALB | MTTP | 0.464 |
| ALB | SLC22A8 | 0.493 |
| ALB | PAH | 0.556 |
| ALB | SLC2A2 | 0.655 |
| ALB | NPHS2 | 0.676 |
| ALDOB | G6PC | 0.904 |
| ALDOB | FABP1 | 0.634 |
| ALDOB | SLC2A2 | 0.626 |
| ALDOB | GSTA1 | 0.456 |
| ALDOB | HAO2 | 0.423 |
| ALDOB | GSTA2 | 0.451 |
| CYP17A1 | CYP4F3 | 0.485 |
| CYP17A1 | CYP4F2 | 0.476 |
| CYP17A1 | CYP3A5 | 0.931 |
| CYP17A1 | REN | 0.468 |
| CYP17A1 | CYP4A11 | 0.468 |
| CYP3A5 | CYP4F3 | 0.403 |
| CYP3A5 | CYP4F2 | 0.433 |
| CYP3A5 | GSTA2 | 0.712 |
| CYP3A5 | GSTA1 | 0.787 |
| CYP3A5 | UGT1A5 | 0.798 |
| CYP3A5 | UGT1A3 | 0.867 |
| CYP3A5 | UGT1A9 | 0.888 |
| CYP3A5 | CYP4A11 | 0.943 |
| CYP4A11 | CYP4F3 | 0.926 |
| CYP4A11 | CYP4F2 | 0.926 |
| CYP4A11 | FABP1 | 0.729 |
| CYP4A11 | PLA2G12B | 0.702 |
| CYP4A11 | UGT1A5 | 0.722 |
| CYP4A11 | UGT1A3 | 0.768 |
| CYP4A11 | UGT1A9 | 0.779 |
| CYP4F2 | CYP4F3 | 0.825 |
| CYP4F2 | PLA2G12B | 0.734 |
| CYP4F3 | PLA2G12B | 0.691 |
| CYP8B1 | SLC10A2 | 0.731 |
| CYP8B1 | G6PC | 0.472 |
| DAO | KMO | 0.427 |
| DAO | GLYAT | 0.607 |
| DAO | HAO2 | 0.95 |
| DAO | PIPOX | 0.955 |
| FABP1 | G6PC | 0.432 |
| FABP1 | REN | 0.499 |
| FABP1 | PAH | 0.401 |
| FABP1 | PLA2G12B | 0.522 |
| FABP1 | SLC2A2 | 0.613 |
| FABP1 | UGT1A9 | 0.722 |
| FABP1 | MTTP | 0.799 |
| FABP1 | GSTA1 | 0.805 |
| G6PC | SLC10A2 | 0.423 |
| G6PC | MTTP | 0.438 |
| G6PC | PAH | 0.451 |
| G6PC | SLC17A3 | 0.583 |
| G6PC | SLC2A2 | 0.761 |
| GLYAT | HPD | 0.427 |
| GSTA1 | GSTA2 | 0.963 |
| HAO2 | TTC36 | 0.551 |
| HAO2 | PIPOX | 0.984 |
| HAO2 | SLC2A2 | 0.595 |
| HAO2 | KMO | 0.59 |
| HAO2 | PLA2G12B | 0.546 |
| HAO2 | PAH | 0.588 |
| HNF4G | SLC17A1 | 0.482 |
| HNF4G | PIPOX | 0.443 |
| HNF4G | SLC2A2 | 0.527 |
| HPD | TTC36 | 0.472 |
| HPD | PAH | 0.738 |
| KMO | PIPOX | 0.426 |
| KMO | SLC2A2 | 0.573 |
| KMO | PAH | 0.632 |
| MIOX | TMEM174 | 0.468 |
| MIOX | UGT1A3 | 0.657 |
| MIOX | UGT1A5 | 0.657 |
| MIOX | UGT1A9 | 0.657 |
| MTTP | SLC6A19 | 0.41 |
| MTTP | SLC2A2 | 0.512 |
| MTTP | PLA2G12B | 0.472 |
| NPHS2 | REN | 0.612 |
| PAH | SLC10A2 | 0.44 |
| PAH | SLC2A2 | 0.685 |
| PAH | PLA2G12B | 0.483 |
| PIPOX | TTC36 | 0.558 |
| PIPOX | PLA2G12B | 0.547 |
| PIPOX | SLC2A2 | 0.556 |
| PLA2G12B | SLC2A2 | 0.663 |
| REN | SLC6A19 | 0.431 |
| SLC10A2 | SLC13A2 | 0.451 |
| SLC10A2 | SLC6A19 | 0.632 |
| SLC13A2 | TMEM174 | 0.412 |
| SLC13A2 | SLC22A13 | 0.461 |
| SLC13A2 | SLC28A1 | 0.443 |
| SLC13A3 | SLC17A3 | 0.429 |
| SLC13A3 | SLC22A11 | 0.479 |
| SLC13A3 | SLC22A13 | 0.499 |
| SLC13A3 | SLC22A7 | 0.515 |
| SLC13A3 | SLC22A6 | 0.658 |
| SLC13A3 | SLC22A8 | 0.682 |
| SLC17A1 | SLC7A9 | 0.603 |
| SLC17A1 | SLC22A7 | 0.563 |
| SLC17A1 | SLC22A13 | 0.708 |
| SLC17A1 | SLC22A6 | 0.726 |
| SLC17A1 | SLC22A8 | 0.747 |
| SLC17A1 | SLC22A12 | 0.876 |
| SLC17A1 | SLC22A11 | 0.881 |
| SLC17A3 | SLC22A11 | 0.82 |
| SLC17A3 | SLC6A19 | 0.66 |
| SLC17A3 | SLC22A13 | 0.757 |
| SLC17A3 | SLC47A2 | 0.446 |
| SLC17A3 | SLC22A8 | 0.762 |
| SLC17A3 | SLC22A7 | 0.69 |
| SLC17A3 | SLC22A12 | 0.851 |
| SLC17A3 | SLC22A6 | 0.749 |
| SLC22A11 | SLC28A1 | 0.482 |
| SLC22A11 | SLC47A2 | 0.603 |
| SLC22A12 | TMEM174 | 0.476 |
| SLC22A12 | SLC47A2 | 0.42 |
| SLC22A13 | SLC7A9 | 0.444 |
| SLC22A13 | TMEM174 | 0.438 |
| SLC22A13 | SLC2A2 | 0.407 |
| SLC22A13 | SLC47A2 | 0.6 |
| SLC22A6 | TMEM174 | 0.401 |
| SLC22A6 | SLC47A2 | 0.688 |
| SLC22A6 | SLC28A1 | 0.431 |
| SLC22A7 | SLC7A9 | 0.468 |
| SLC22A7 | SLC2A2 | 0.565 |
| SLC22A7 | SLC47A2 | 0.625 |
| SLC22A7 | SLC22A8 | 0.851 |
| SLC22A7 | SLC28A1 | 0.619 |
| SLC22A8 | SLC7A9 | 0.455 |
| SLC22A8 | TMEM174 | 0.425 |
| SLC22A8 | SLC47A2 | 0.775 |
| SLC22A8 | SLC28A1 | 0.525 |
| SLC2A2 | SLC6A19 | 0.428 |
| SLC2A2 | TTC36 | 0.447 |
| SLC47A2 | SLC6A19 | 0.487 |
| SLC6A19 | SLC7A9 | 0.757 |
| UGT1A3 | UGT1A9 | 0.416 |
|  |  |  |


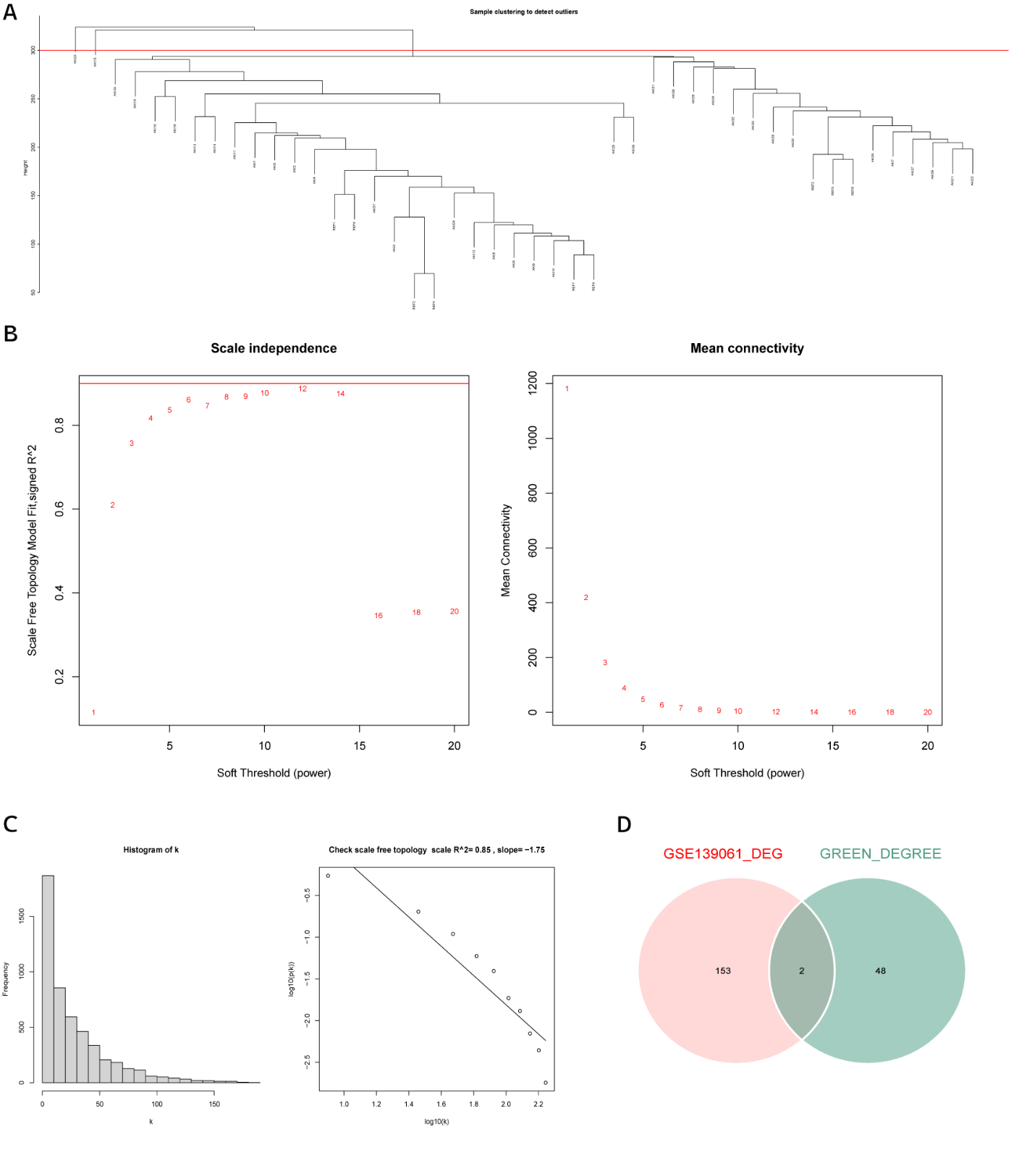


**Supplementary Figure 1.** (A)Sample clustering to detect outliers. (B)Analysis of the scale-free fit index and mean connectivity for the Soft threshold power (β) in Weighted correlation network analysis. (C) The histogram of connectivity distribution and the scale-free topology. (D)Hub genes were selected based on overlap between PPI and the differentially expressed genes in the dataset of GSE139061.
